# Assessment of autosomal dominant polycystic kidney disease severity using deep learning–based kidney volumetry in CT

**DOI:** 10.1186/s41747-026-00840-w

**Published:** 2026-11-12

**Authors:** Konstantinos Koukoutegos, Tom Oyen, Raymond Oyen, Frederik De Keyzer, Frederik Maes, Hilde Bosmans

**Affiliations:** 1 Department of Imaging and Pathology, Division of Medical Physics, KU Leuven, Leuven, Belgium; 2 Department of Radiology, UZ Leuven, Leuven, Belgium; 3 Department of Electrical Engineering, ESAT/PSI, KU Leuven, Leuven, Belgium; 4 Present Address: Department of Pathology and Laboratory Medicine, Division for Computational Pathology, Indiana University School of Medicine, Indianapolis, IN USA

**Keywords:** Abdomen, Deep learning, Kidney, Polycystic kidney (autosomal dominant), Tomography (x-ray computed)

## Abstract

**Objectives:**

Accurate quantification of total kidney volume (TKV) and Mayo imaging classification (MIC) is essential for assessing disease severity in autosomal dominant polycystic kidney disease (ADPKD). This study aimed to develop and validate a deep learning–based method using noncontrast computed tomography (CT) scans to automate TKV measurement and MIC assignment.

**Materials and methods:**

A retrospective dataset of 60 ADPKD patients was used to train a three-dimensional (3D) UNet model for kidney segmentation and TKV estimation. Kidney axes were calculated using principal component analysis (PCA). The model was tested on consecutive scans from 30 patients (median interval: 3.6 years) and 28 external patient scans. Segmentation accuracy was evaluated using the Dice similarity coefficient (DSC), and measurement reliability was assessed using the intraclass correlation coefficient (ICC). MIC was assigned and compared across timepoints.

**Results:**

The model achieved high segmentation accuracy (DSC: 0.93 ± 0.04 [mean ± standard deviation] and 0.94 ± 0.03 for consecutive scans; 0.96 ± 0.08 for external validation). Manual and automated TKV measurements were strongly correlated (ICC: 0.94–0.99, *p* < 0.001), with a mean volume error of 7.61 ± 7.26%. MIC assignment matched in 90–93.3% of cases. Kidney dimensions (length > 16.5 cm, width > 9.5 cm, depth > 8 cm) predicted high-risk MIC classes with positive predictive values > 88%. Longitudinal analysis showed significant increases in TKV and declines in estimated glomerular filtration rate–eGFR (*p* < 0.001).

**Conclusion:**

The proposed method enables accurate TKV quantification and MIC classification from noncontrast CT, supporting severity assessment and longitudinal monitoring in ADPKD.

**Key Points:**

***Question*** Can a deep learning method using noncontrast CT accurately automate total kidney volume measurement and Mayo Imaging Classification assignment in autosomal dominant polycystic kidney disease?

***Findings*** In internal and external testing, automated kidney segmentation showed high agreement with reference measurements and correctly assigned Mayo Imaging Classification in most cases with overall accuracy.

***Relevance statement*** Automated analysis of noncontrast CT provided reliable kidney volume quantification and disease severity classification, reducing manual workload while enabling longitudinal assessment of disease progression in autosomal dominant polycystic kidney disease.

**Graphical Abstract:**

**Figure:**
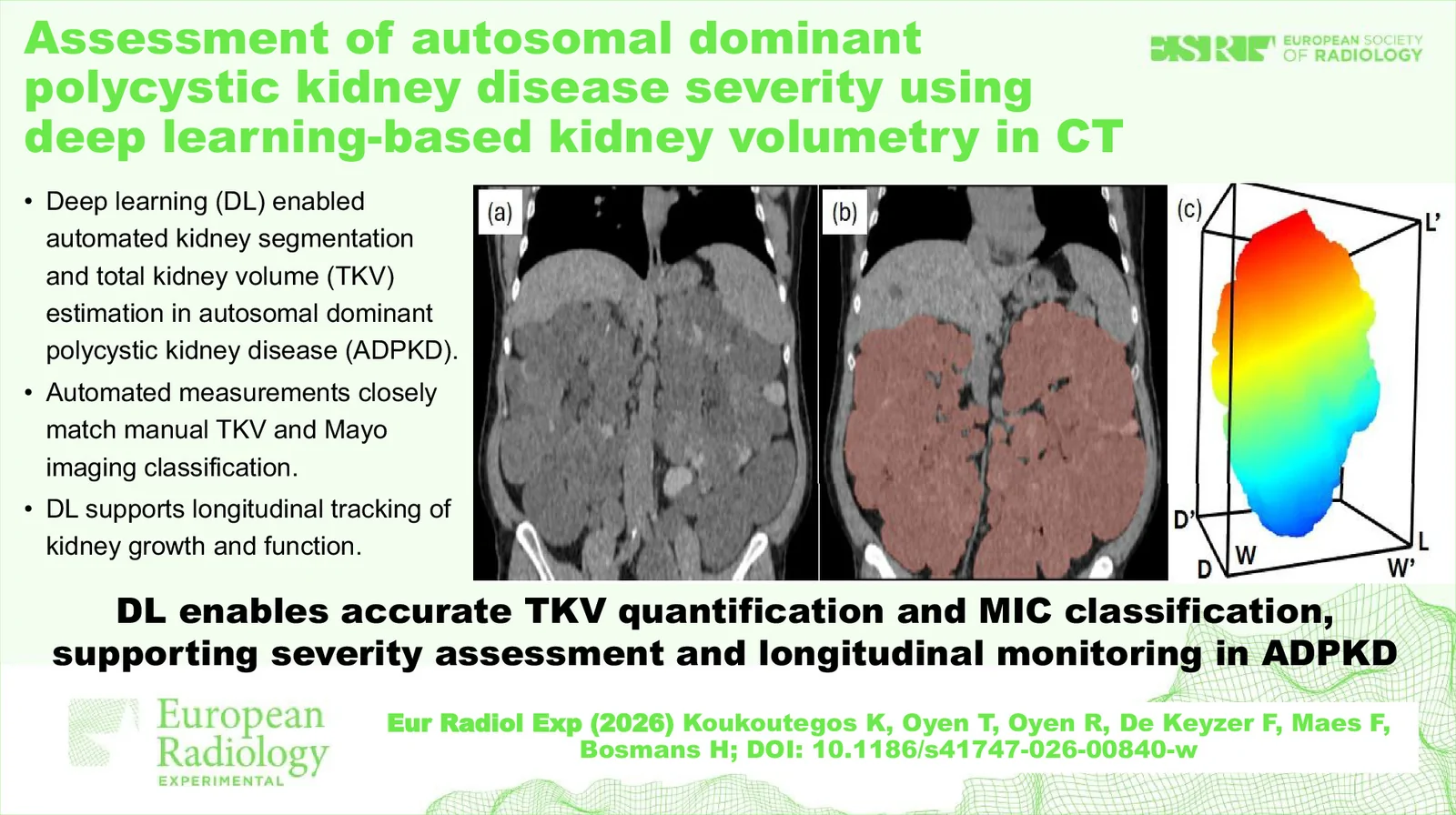

## Introduction

Autosomal dominant polycystic kidney disease (ADPKD) is the most common hereditary renal disease and the fourth leading cause of renal failure in adults [1, 2]. Although relatively common among inherited kidney disorders, ADPKD is classified as a rare disease according to the National Organization for Rare Disorders (NORD, https://rarediseases.org/). ADPKD is most commonly associated with mutations in PKD1 and PKD2 genes, affecting between 1 in 1,000 and 1 in 2,500 individuals [3–5]. The disease course is characterized by the development and expansion of fluid-filled cysts, distributed across the renal cortex and medulla. While the disease is initially relatively stable, a gradual decline in renal function is observed and evolves over many decades, as the growing cysts replace the functional parenchyma. Later disease stages are characterized by a period of accelerated functional decline, leading to end-stage renal disease in up to 50% of the patients [6, 7]. ADPKD progression is slow during the first decades, and early renal function assessments by means of serum creatinine and glomerular filtration rate (GFR) measurements pp are poor prognosticators, as they remain within normal ranges until the late clinical course [8].

The development of new cysts and the progressive enlargement of existing ones drive the volume of the kidneys to increase. The Consortium for Radiologic Imaging Studies of PKD (CRISP) has demonstrated that height-adjusted total kidney volume (HtTKV) predicts the risk of GFR decline [9], paving the way for the U.S. Food and Drug Administration and the European Medicines Agency to qualify TKV as a prognostic biomarker of ADPKD [10]. In line with these observations, the Mayo imaging classification (MIC) [11] is a risk stratification tool that utilizes the HtTKV, indexed for age and measured at a single time point, to determine renal outcomes in patients with class 1 ADPKD, *i.e*., typical, bilateral, and diffuse distribution of cysts. The MIC assigns patients into 5 risk classes (1A–1E) based on the annual kidney growth rate: 1A: < 1.5%, 1B: 1.5–3%, 1C: 3–4.5%, 1D: 4.5–6%, and 1E: > 6%. High-risk (1C–1E) patients may be candidates for specific clinical trials or become eligible for tolvaptan treatment [12], while classification in 1D–1E categories may be an indication for nephrectomy [13].

The straightforward approach to measure the TKV is by manual planimetry, where both kidney contours are manually delineated across a 3D imaging stack, usually acquired with either magnetic resonance imaging (MRI) or computed tomography (CT). Although this method is the reference standard, it is labor-intensive and subject to inter- and intra-observer variability even in normal-sized healthy kidneys [14, 15]. Semiautomated approaches, including stereology and ellipsoid formulas [11, 16–18], have been promoted to reduce the occupational time, but their agreement compared to the reference standard is suboptimal [16, 17]. The advent of deep learning (DL) enabled fully automated methods, primarily focused on normal kidneys [19–21], while the Kidney and Kidney Tumor Segmentation challenge (KiTS) [22] stimulated the development of robust methods for kidneys with pathologies [23–26]. However, KiTS consists of contrast-enhanced CT scans, which may be unsuitable in clinical settings where the administration of contrast is not indicated or contraindicated, such as in patients with impaired renal function [27].

In the context of polycystic kidneys, automated methods based on deep learning (DL) have also been introduced, primarily based on MRI [28–33]. MRI is generally preferred because of its high tissue contrast and lack of ionizing radiation, allowing long-term follow-up. However, CT offers shorter acquisition times, higher resolution, more standardized protocols across scanners, and lower costs compared to MRI [34], making the assessment of TKV and HtTKV from CT images relevant in many situations. Existing automated methods for ADPKD have largely focused on automating TKV estimation alone. In contrast, our proposed framework additionally provides automated calculation of the renal axes and MIC within a fully automated pipeline. The goal of this study was to develop and evaluate a DL-based method for automated kidney segmentation and volumetric analysis using CT in patients with ADPKD, including TKV, HtTKV, renal axes, and MIC-based severity assessment.

## Materials and methods

### Dataset

**Fig. 1:**
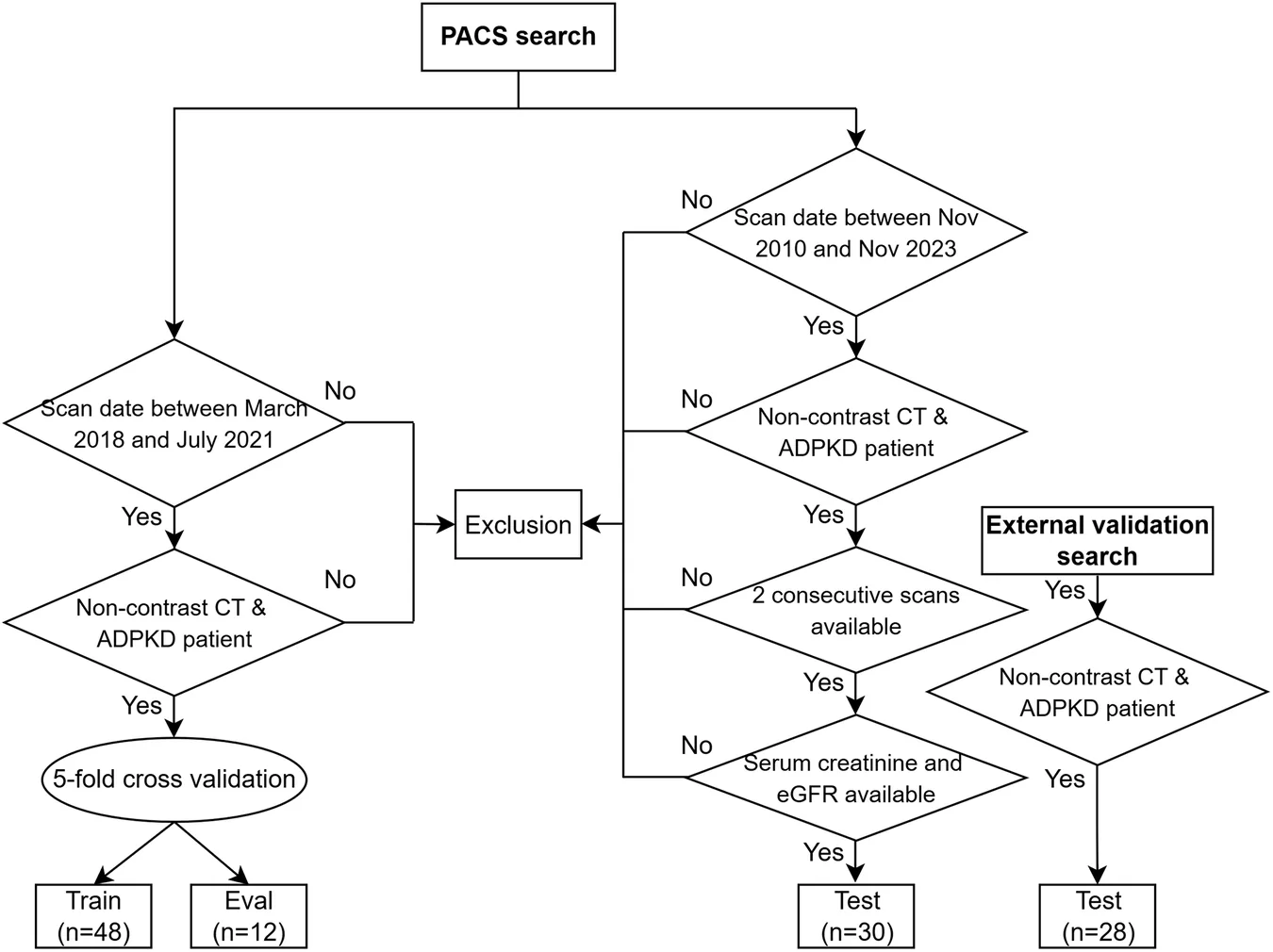
Study inclusion process. ADPKD, Autosomal dominant polycystic kidney disease; CT, Computed tomography; eGFR, Estimated glomerular filtration rate

This retrospective study included patients diagnosed with ADPKD who underwent noncontrast CT at our center for follow-up of TKV. The dataset was divided into two groups: a training group and a test group. The training group comprised 60 individuals who had a CT scan between March 2018 and July 2021, while the test group consisted of 30 patients who had two consecutive abdominal CT scans between November 2010 and November 2023. This retrospective study involving human participants was approved by the local institutional ethics committee of UZ Leuven (University Hospital Leuven) (protocol number S68487). The requirement for informed consent was waived due to the retrospective nature of the study. We additionally used a dataset of 28 ADPKD cases from an external hospital (OLV Hospital Aalst) for validation purposes. A detailed description of the scanning protocol along with relevant dataset characteristics are available in the Supplementary Material, while the study inclusion process is depicted in Fig. 1. Manual segmentation of the left and right kidneys for both the training and test groups was carried out using the SegmentEditor module of 3D Slicer [35]. A junior radiologist with 5 years of experience performed the segmentations under the supervision and guidance of a senior radiologist with four decades of expertise. The external validation set cases were manually segmented by the same radiologist who segmented our internal test set of 30 patients. The manually labeled volumes included solely the cysts and renal parenchyma, excluding the renal sinus fat and hilum. Total kidney volume and kidney axes (length, width, depth) were calculated based on the manual labels for all subjects, while manual MIC was calculated for each of the two consecutive scans of the entire test set. Anthropomorphic parameters (patient age and height) and serum creatinine measurements were extracted from the electronic health record. The CKD-EPI equation [36] was utilized to calculate the estimated GFR (eGFR).

### Kidney segmentation

The training cohort was split into training and validation subgroups, in a 5-fold cross-validation setting, with ratios of 0.8 and 0.2, respectively. The CT scans were resampled using trilinear interpolation to an isotropic voxel spacing of 1.75 mm, and the pixel intensities were truncated using a window level/width of 50/150 HU (identical to the level/width used during the manual annotation process), followed by min-max normalization to [0,1]. The window level choice was based on the kidney HU distribution of the entire training cohort. The manual segmentations were transformed into one-hot encoded representations with three classes, *i.e*., background, right, and left kidney.

**Fig. 2:**
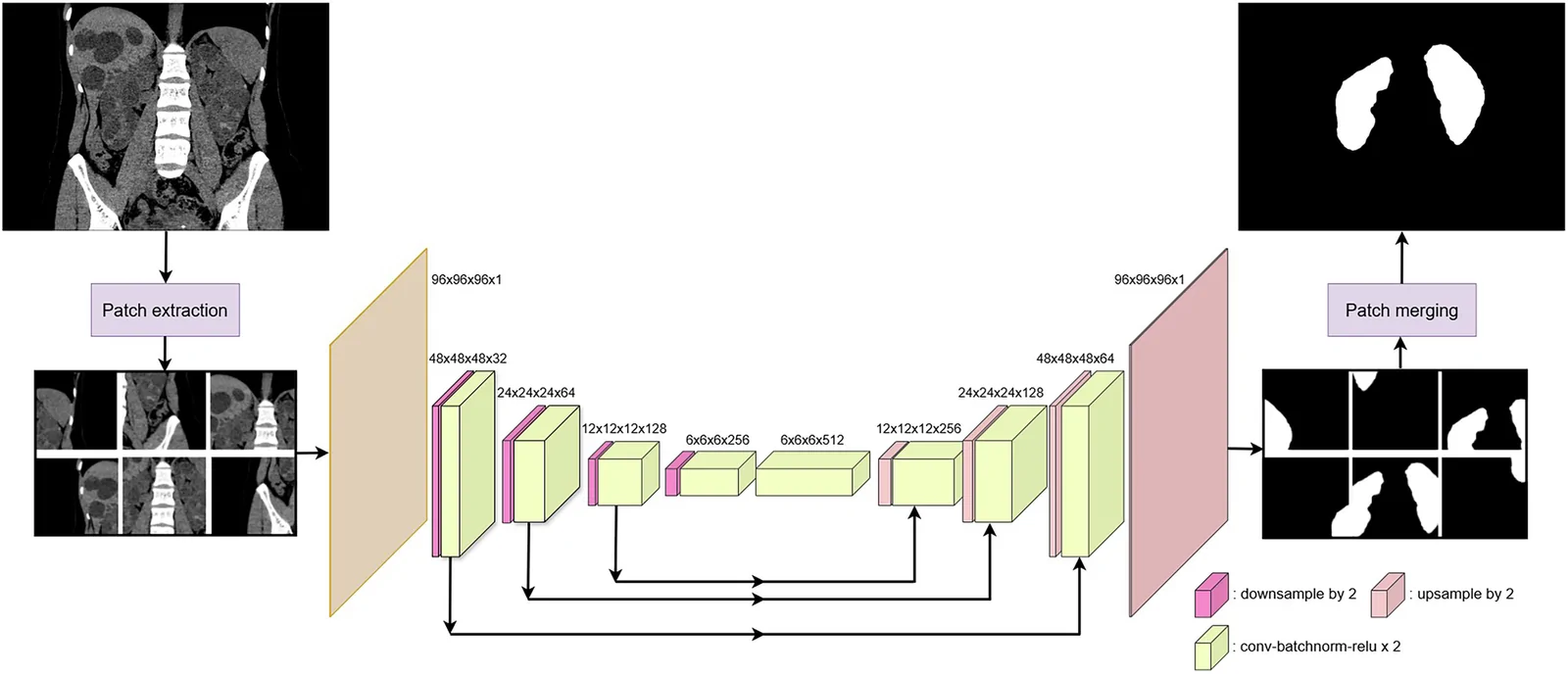
UNet architecture for kidney segmentation. Image patches of size 96 × 96 × 96 are extracted from the initial computed tomography volume and downsampled by a factor of 2 in each encoding layer. The reverse process takes place at the decoding path. The final segmentation is obtained by merging the predictions for different patches

The preprocessed images were used to train a 3D UNet [37] convolutional neural network to automatically segment both kidneys. The architecture was based on Kerfoot et al [38], with both the encoding and decoding paths comprising residual connections to minimize vanishing gradients. Extensive augmentation techniques were utilized to artificially extend the number of training samples, including the addition of Gaussian noise, random intensity shifting, random flips in x, y, and z axes, and random image patch crop of size 96x96x96. We used the Adam [39] optimizer to adjust the network’s parameters, using a learning rate of 3 × 10^-4^. A cosine annealing with warm restarts scheduler reduced the learning rate to 10^-5^ after the first 300 epochs, with a period of T = 100. The Dice loss [40] was used as objective criterion, and a dropout rate [41] of 20% was applied to avoid overfitting. Images were processed in batches of 10, with each sample consisting of an image patch, cropped randomly from the initial CT, ${Pi}\in {{\mathbb{R}}}^{96x96x96},i=\left\{1,\ldots ,10\right\}.$ Batch normalization was applied in order to normalize the features across batches. During inference, cropped patches were predicted separately and merged to obtain the final complete segmentation map. The network architecture and training were implemented using PyTorch v1.10.0 (https://pytorch.org/) and MONAI v1.0.1 (https://monai.io/), while the overall framework is depicted in Fig. 2. Experiments were executed on a single NVIDIA GeForce RTX 3,090 with 24 GB for 2,500 epochs. The training took approximately 12.5 h, while inference time was about 16–18 s on average per CT scan. For evaluation purposes, we selected a single model that performed the best across the 5-fold cross-validation setting.

### Kidney measurements

Quantitative measurements were obtained by post-processing of the segmented kidneys. The TKV was calculated by combining the right and left kidney volumes, while HtTKV (in mL/m) was obtained by dividing TKV (in mL) by patient height (in m). Additionally, the kidney size along each axis (*i.e*., length, width, and depth) was calculated for each kidney separately using principal component analysis (PCA) of the 3D point cloud of kidney voxels. The mean kidney length (KL), width (KW), and depth (KD) were obtained by averaging the values of both kidneys. Height-adjusted values HtKL, HtKW, and HtKD were obtained similarly to HtTKV. The DL-based MIC assignment was calculated by indexing the HtTKV for patient age, using the online tool (https://www.mayo.edu/research/documents/pkd-center-adpkd-classification/doc-20094754).

### Baseline TotalSegmentator comparison

To establish a baseline segmentation performance using a state-of-the-art model, we evaluated TotalSegmentator [42], an open-source deep learning framework for automated whole-body organ segmentation. The official GitHub implementation was used without fine-tuning on ADPKD-specific data. Kidney segmentations were generated using the 1.5 mm isotropic resolution model, selected to ensure a fair comparison with our in-house framework. Performance evaluation was conducted on the in-house test dataset comprising 60 CT examinations obtained from 30 patients with ADPKD.

### Statistical analysis

Segmentation performance was evaluated in terms of Dice Similarity Coefficient (DSC), where a DSC of 1 indicates perfect overlap of the manual and automated segmentation, while a DSC of 0 implies no overlap. Additionally, the Hausdorff distance (in mm) was used to evaluate the agreement between the manual ground truth and automated DL-based kidney contours. The intraclass correlation coefficient (ICC) was used to estimate the reliability of automated *versus* manual volume and size measurements using a 2-way mixed effects model based on a single rater making consistent measurements. ICC values can be interpreted as [43]: poor (ICC < 0.50); moderate (0.50 ≤ ICC ≤ 0.75); good (0.75 ≤ ICC ≤ 0.90); and excellent (ICC ≥ 0.90). Bland–Altman analysis was used to compare manual and DL-based TKV measurements. MIC class accuracy was determined as the number of correct MIC predictions compared to the manual MIC assignment. Statistical analysis was performed using Python v3.9.18, with SciPy v1.9.1. ICC values were calculated using Pingouin v0.5.3. Normally distributed continuous variables were expressed by their mean and standard deviation (SD), while non-normally distributed variables were expressed as medians and interquartile ranges. Data normality was tested using the Shapiro–Wilk test, and paired *t*-tests were used to compare normally distributed variables. Pearson’s correlation was used to assess the relationship of different variables. Categorical variables were expressed as absolute counts and percentages, and the Wilcoxon signed-rank test was used to assess statistical significance. A *p*-value less than 0.001 was considered statistically significant.

## Results

### Dataset characteristics

Table 1 demonstrates the clinical characteristics of the study test set, comprising 30 patients with 2 consecutive CT scans, with a median interval time of 3.6 years. eGFR showed a statistically significant difference between the two consecutive scans (*p* < 0.001), with the mean value decreasing from 75.50 ± 35.82 mL/min/1.73 m² in the first scan to 66.16 ± 40.35 mL/min/1.73 m² in the second scan. Serum creatinine had an increase with an average difference of 0.58 mg/dL (*p* < 0.001). As expected for this pathology, the TKV, as quantified by manual delineation, significantly increased as well, with mean values of 1,741 ± 1,479 mL and 2,351 ± 2,431 mL (*p* < 0.001), respectively. Manual measurements of KL, KW, and KD were all statistically significantly different between the scans (*p* < 0.001), with average differences of 13.8 mm, 9.0 mm, and 4.5 mm, respectively. MIC determined by manual TKV assessment, assigned subjects to both low- and high-risk classes (*i.e*., 1B–1E) with Class 1B (*n* = 8, 26.6%), Class C (*n* = 8, 26.6%), Class D (*n* = 6, 20%), and Class 1E (*n* = 8, 26.6%). MIC distribution did not differ significantly between the scans, although one subject was assigned to 1 MIC class higher, while another subject was assigned to 1 MIC class lower.

**Table 1:** Study test set patient characteristics

Test cohort, *n* = 30	Scan 1	Scan 2
Age*, years	38.11 (28.51–49.49)	41.79 (31.25–50.65)
Sex, men:women	19:11	19:11
Height*, m	1.74 (1.71–1.83)	1.74 (1.71–1.83)
Weight*, kg	74.7 (68.7–90.6)	76.5 (66–94.77)
Serum creatinine^#^, mg/dL	1.34 (0.66)	1.92 (1.52)
eGFR^#^, mL/min/1.73 m^2^	75.50 (35.82)	66.16 (40.35)
TKV^#^, mL	1,741.75 (1,479.79)	2,351.12 (2,431.46)
KL^#^, mm	184.87 (42.05)	198.66 (49.97)
KW^#^, mm	111.99 (29.40)	120.96 (38.13)
KD^#^, mm	95.22 (29.72)	99.75 (34.62)
MIC (manual)		
1A	0 (0%)	0 (0%)
1B	8 (26.6%)	8 (26.6%)
1C	8 (26.6%)	8 (26.6%)
1D	6 (20%)	6 (20%)
1E	8 (26.6%)	8 (26.6%)

Values with * are presented as median (interquartile ranges); values with ^#^ are presented as mean (standard deviation); MIC is presented as absolute number (percentage)

*eGFR* Estimated glomerular filtration rate, *KD* Kidney depth, *KL* Kidney length, *KW* Kidney width, *MIC* Mayo imaging classification, *TKV* Total kidney volume

### Segmentation performance

**Fig. 3:**
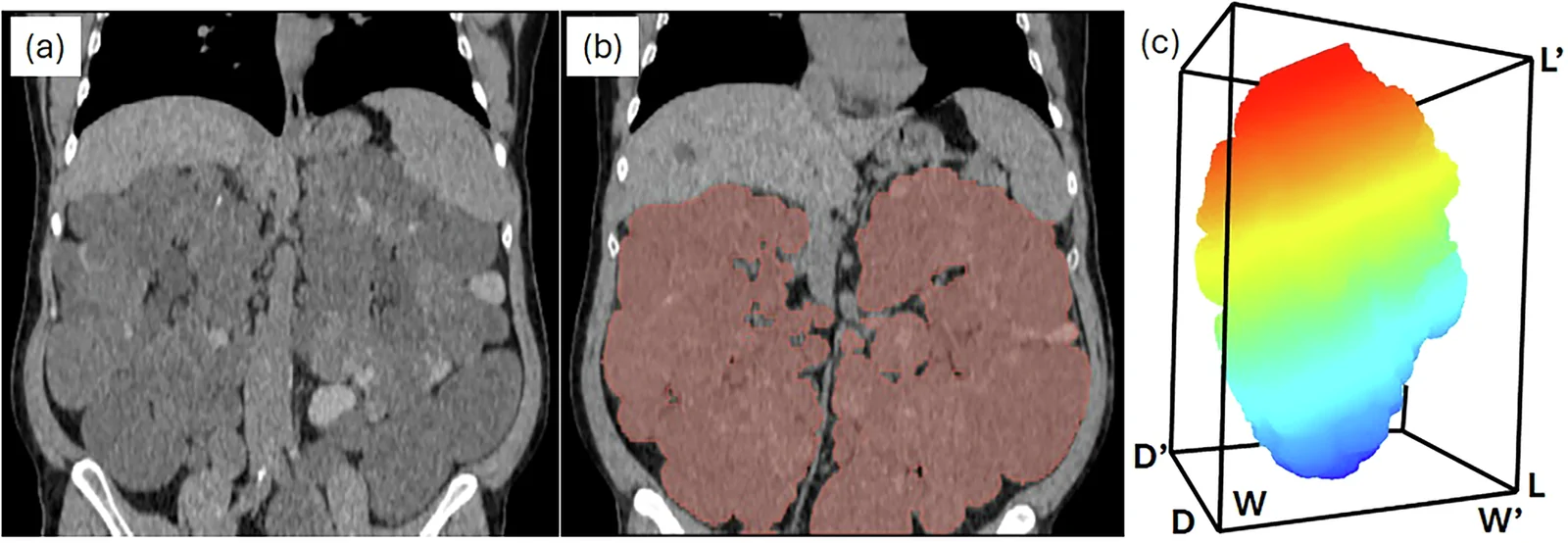
**a** CT image. **b** Automated kidney segmentation. **c** Three-dimensional point-cloud of kidney voxels and its object-oriented bounding box calculated using principal component analysis (PCA)

The segmentation performance of the automated method demonstrated a high DSC for both scans, with an average of 0.93 ± 0.04 for scan 1 and 0.94 ± 0.03 for scan 2. The average Hausdorff distance was 29 ± 24 mm for scan 1 and 28 ± 26 mm for scan 2. The difference in DSC and Hausdorff distance between both scans was not statistically significant (*p* = 0.35 and *p* = 0.88, respectively). Figure 3a, b demonstrates a CT image and its corresponding automated segmentation, while the renal axes calculation for a single kidney volume is illustrated in Fig. 3c.

### External validation set

The segmentation performance of the automated method in the external validation set demonstrated a high DSC, averaging 0.96 ± 0.08 and an average Hausdorff distance of 7 ± 22 mm (mean ± standard deviation). The mean percentage TKV error was 8.1 ± 29.9%. The absolute errors in measuring the KL, KW, and KD were 6.3 ± 19.3 mm, 4.4 ± 14.0 mm, and 3.1 ± 9.3 mm, respectively.

### Comparison of manual *versus* automated measurements

A quantitative comparison of manual and automated kidney measurements for either scan is summarized in Table 2. For scan 1, the ICC (95% CI, *p*-value) for TKV obtained with automated *versus* manual segmentation was 0.97 (0.95–0.99, *p* < 0.001), while the ICCs for KL, KW, and KD were 0.94 (0.90–0.98, *p* < 0.001), 0.97 (0.94–0.99, *p* < 0.001), and 0.97 (0.94–0.99, *p* < 0.001) respectively. The mean percentage volume error was 7.6 ± 7.2%. The mean absolute errors in KL, KW, and KD measurement were 4.7 ± 6.3 mm, 3.6 ± 5.0 mm, and 6.9 (4.0–9.1) mm, respectively. The MIC based on the automated segmentation agreed with the manual MIC in 27 of the 30 cases (90%), overestimating severity by 1 MIC class in 2 of the 30 cases (6.66%).

**Table 2:** Quantitative comparison between manual and deep learning–based measurements

Parameter	Scan 1	Scan 2
DSC	0.93 ± 0.04	0.94 ± 0.03
Hausdorff distance, mm	29.12 ± 24.13	28.68 ± 26.76
MPVR		
TKV, %	7.61 ± 7.26%	7.24 ± 8.44%
MAE		
KL, mm	4.75 ± 6.38	3.79 ± 4.58
KW, mm	3.69 ± 5.01	4.46 ± 5.01
KD, mm	6.90 (4.02–9.19)	3.06 ± 3.45
ICC		
TKV	0.97 (0.95–0.99, *p* < 0.001)	0.96 (0.93–0.98, *p* < 0.001)
KL	0.94 (0.90–0.98, *p* < 0.001)	0.97 (0.94–0.99, *p* < 0.001)
KW	0.97 (0.94–0.99, *p* < 0.001)	0.98 (0.96–0.99, *p* < 0.001)
KD	0.97 (0.94–0.99, *p* < 0.001)	0.99 (0.99–1.0, *p* < 0.001)
MIC correct assignment	27/30 (90%)	28/30 (93.3%)
IT, s	15.42 ± 6.40	18.52 ± 8.87

Data are presented as mean ± standard deviation or median (interquartile range)

*DSC* Dice similarity coefficient, *ICC* Intraclass correlation coefficient, *IT* Inference time, *KD* Kidney depth, *KL* Kidney length, *KW* Kidney width, *MAE* Mean absolute error, *MPVR* Mean percentage volume error, *TKV* Total kidney volume

For scan 2, the ICC values for TKV, KL, KW, and KD were 0.96 (0.93–0.98, *p* < 0.001), 0.97 (0.94–0.99, *p* < 0.001), 0.98 (0.96–0.99, *p* < 0.001), and 0.99 (0.99–1.0, *p* < 0.001), respectively. The mean percentage volume error was 7.2 ± 8.4%, and the absolute errors in KL, KW, and KD were 3.7 ± 4.5 mm, 4.4 ± 5.0 mm, and 3.0 ± 3.4 mm, respectively. The MIC based on the automated segmentation agreed with the manual MIC in 28 of the 30 cases (93.3%) and overestimated severity by 1 MIC class in 2 of the 30 cases (6.6%).

**Fig. 4:**
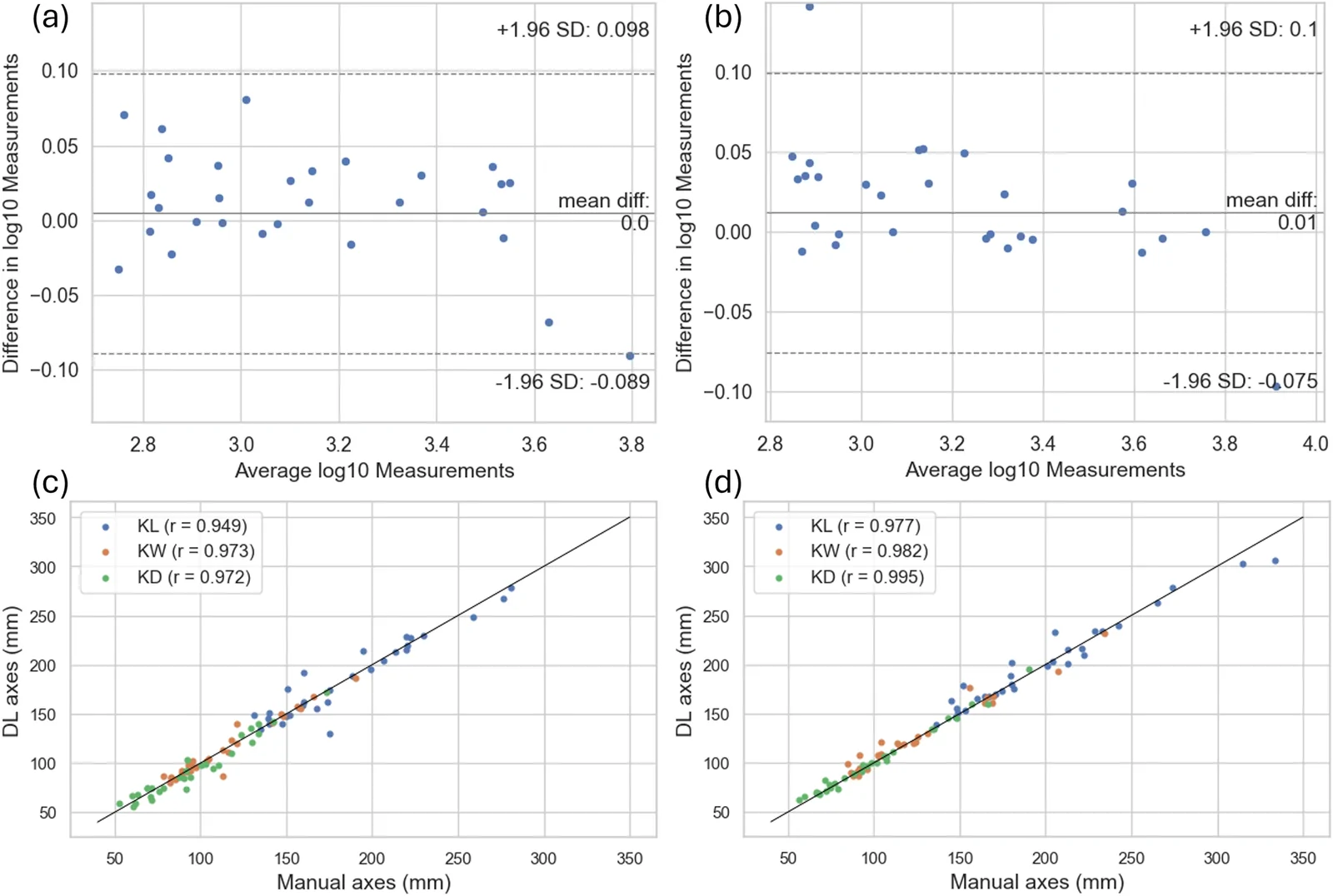
Bland–Altman analysis for the total kidney volume (TKV) agreement of the first (**a**) and second (**b**) scans (in log scale). Renal axes correlation between manually and DL-derived length (KL), width (KW), and depth (KD) for the first (**c**) and second (**d**) scans. Pearson’s *r* values are provided in the legend inside each panel

The Bland–Altman analysis for manual *versus* automated TKV and the correlation between manual *versus* automated kidney sizes are depicted in Fig. 4 for scan 1 and scan 2 separately.

### Comparison of kidney measurements between consecutive scans

**Fig. 5:**
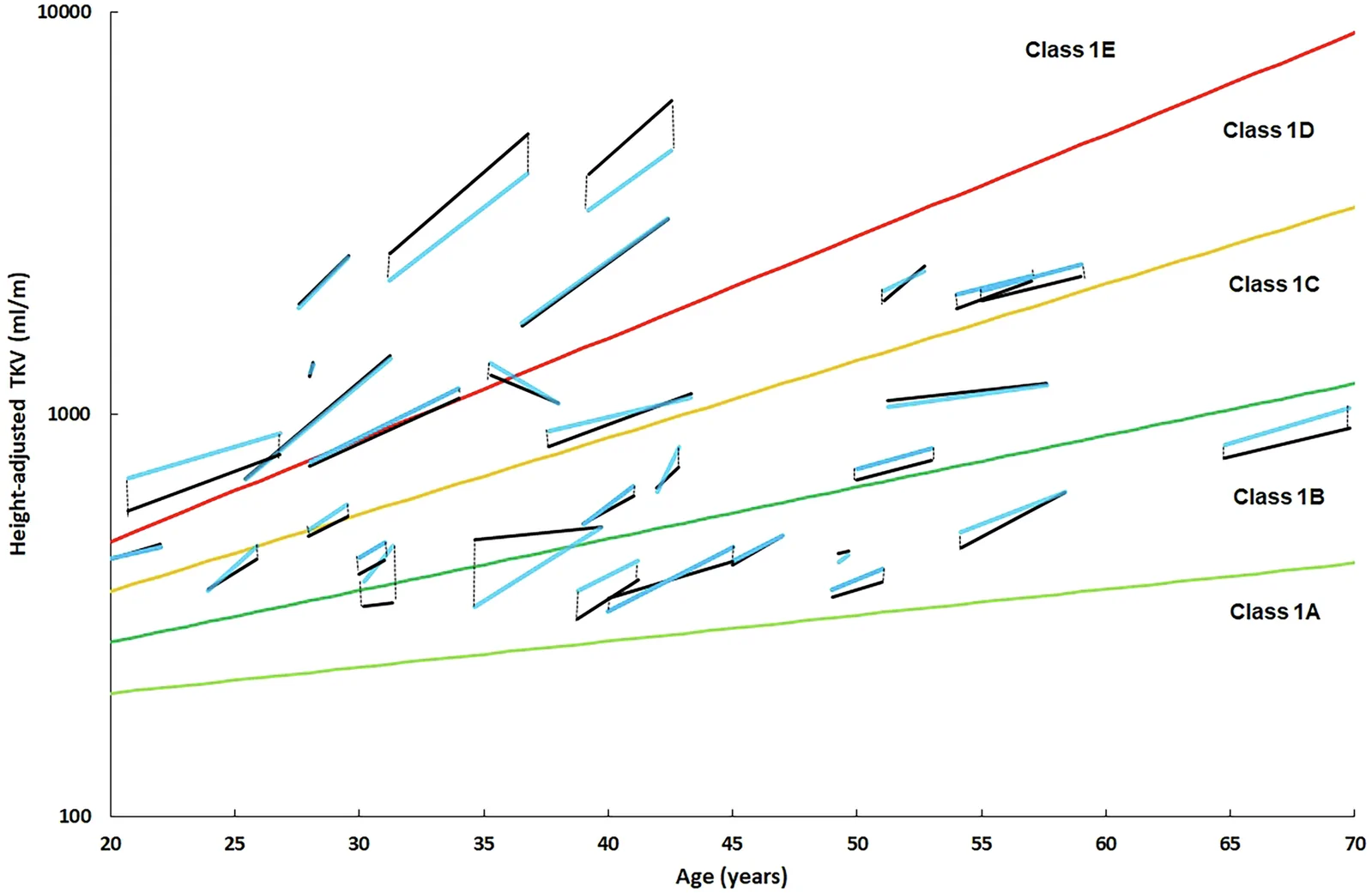
Manual (black) and automated (blue) Mayo imaging classification (MIC) assignment. The automated approach closely follows the manual assignment in most cases, even when the MIC dropped by 1 class in a single case

Similar to the manual measurements, automated measurements of TKV resulted in statistically significant differences between the two scans (*p* < 0.001), with an average difference of 609.3 mL for the manual and 534.0 mL for the automated measurement. Automated measurements of KL, KW, and KD all differed significantly (*p* < 0.001) with mean differences of 15.1 mm, 10.5 mm, and 7.4 mm, respectively. The change in MIC between scan 1 and scan 2 is shown in Fig. 5 for both the manual and the automated approach. Automated assessment of MIC did not result in statistically significant differences between the two scans, as for manual assessment (*p* > 0.001).

### MIC prediction based on kidney length, width, and depth

We investigated the predictive performance of KL, KW, and KD in determining high-risk MIC classes 1C–1E. For patients aged < 45 years, KL > 16.5 cm yielded a positive predictive value (PPV) of 91% (95% CI: 62–98%) for scan 1 and 100% (95% CI: 78–100%) for scan 2, while KW > 9.5 cm and KD > 8 cm yielded PPVs of 100% for both scans. In comparison, when the entire patient population was included, the MIC predictive power of the kidney sizes degraded: the same criteria for KL, KW, and KD had PPVs of 88%, 84%, and 83%, respectively, for scan 1 and 90%, 90%, and 89%, respectively, for scan 2 (Table 3).

**Table 3:** MIC high-risk 1C–1E prediction by mean kidney length (KL > 16.5 cm), width (KW > 9.5 cm), and depth (KD > 8 cm)

Scan	Test cohort	Measurement	Accuracy (%)	Sensitivity (%)	Specificity (%)	PPV (%)	NPV (%)
1	30^d1^	KL	70	68 (47–84)	75 (41–93)	88 (66–97)	46 (23–71)
		KW	77	73 (52–87)	87 (53–98)	94 (73–99)	54 (29–77)
		KD	67	68 (47–84)	62 (31–86)	83 (61–94)	42 (19–68)
	20^d2^	KL	60	59 (36–78)	66 (21–94)	91 (62–98)	22 (6–55)
		KW	70	65 (41–83)	100 (44–100)	100 (74–100)	33 (12–65)
		KD	65	59 (36–78)	100 (44–100)	100 (72–100)	30 (11–60)
2	30^d1^	KL	83	86 (67–95)	75 (41–93)	90 (71–97)	67 (35–88)
		KW	80	82 (61–93)	75 (41–93)	90 (70–97)	60 (31–83)
		KD	73	73 (52–87)	75 (41–93)	89 (67–97)	50 (25–75)
	19^d2^	KL	84	82 (59–94)	100 (34–100)	100 (78–100)	40 (12–77)
		KW	79	76 (53–90)	100 (34–100)	100 (77–100)	33 (10–70)
		KD	68	65 (41–83)	100 (34–100)	100 (74–100)	25 (7–59)

The values in parentheses represent 95% confidence intervals

*d1* Entire study test set, *d2* Subgroup of patients < 45 years, *KD* Kidney depth, *KL* Kidney length, *KW* Kidney width, *MIC* Mayo imaging classification, *NPV* Negative predictive value, *PPV* Positive predictive value

### TotalSegmentator performance

Evaluation of TotalSegmentator on the in-house ADPKD test dataset demonstrated a median DSC of 0.45 [0.14, 0.76] for kidney segmentation. Although the model had been trained on datasets that partly contained kidneys with cystic alterations, the substantial morphologic variability and extensive cystic burden characteristic of ADPKD were likely underrepresented during its training. Comparison of the DSC distribution between our proposed method and TotalSegmentator was statistically significant (*p* < 0.001). Figure S1 in the Supplementary Material demonstrates the DSC distributions of both methods.

## Discussion

In this study, we developed and validated a 3D UNet-based approach to segment kidneys on noncontrast CT and extract TKV and renal axes in the radiological evaluation and follow-up of patients with ADPKD. Comparison of the automated approach to the manual ground truth in 30 patients demonstrated promising results. The 3D UNet generalizability was further evaluated using an independent test set from an external institution.

For each patient of our internal test set, 2 scans with a median interval time of 3.6 years were available. The average DSC (0.93 and 0.94) indicated a high level of agreement between the automated and manual kidney segmentations, while the average Hausdorff distance (29 mm and 28 mm) was influenced by outlier predictions of very large kidneys and highlighted that our approach is sensitive to the kidney size. Although ICC for both the TKV and the renal axes (KL, KW, and KD) showed excellent reliability (> 0.90) between automated and manual measurements, for both scans, our Bland–Altman analysis demonstrates a clear proportional bias as the mean difference widens substantially for large TKV values. Both the high Hausdorff distance and this bias could potentially limit the direct clinical applicability of the proposed method in cases of large kidneys. Automated MIC assessment had an accuracy of 90% for scan 1 and 93.3% for scan 2, with overall 4 out of 60 scans being assigned 1 MIC class higher and 1 of 60 scans 1 MIC class lower by the automated approach. This underestimation of 1 MIC class compared to the manual approach was caused by an erroneous segmentation, where the UNet missed a part of the kidney located close to the liver. Interestingly, there was one case for which both manual and automated MIC assignment dropped 1 class between the consecutive scans. Clinical review revealed that this patient had undergone a gastric sleeve operation between scan 1 and scan 2, which likely caused the kidneys to decrease in size, resulting in a lower MIC assignment, something that the automated approach was able to capture.

MIC prediction based on mean kidney axes revealed results similar to the CRISP cohort and Akbari et al [17, 44]. In young patients aged < 45 years, KL > 16.5 cm can predict high-risk MIC 1C–1E with high certainty (PPV of 91% for scan 1 and 100% for scan 2). Furthermore, in our study, we showed that KW > 9.5 cm or KD > 8 cm demonstrated improved certainty with PPVs of 100% for both scans of the consecutive sequence. The predictive performance of MIC based on kidney axes dropped when the entire test cohort, including older patients, was considered.

Validation in an external dataset of 28 patients demonstrated good generalizability, indicating that the proposed method can be safely applied across different hospitals, different imaging devices, and varying scanning protocols.

Between-scan comparison indicated a significant difference in renal function as measured by eGFR and serum creatinine. Both manual and automated CT-based measurements demonstrated significant differences between scan 1 and scan 2. Similar trends indicate that the automated approach is sensitive enough in detecting subtle anatomical changes that can be associated with functional impairment. No significant changes were found in the MIC assignment between the two consecutive scans.

As a baseline comparison, we evaluated the publicly available TotalSegmentator on the in-house ADPKD dataset. Although TotalSegmentator is a robust state-of-the-art segmentation model trained on a large dataset, its performance substantially decreased in the presence of severe cystic renal deformation. This finding highlights that generic segmentation frameworks, while highly effective for anatomically normal or mildly altered organs, may not sufficiently capture the extensive morphologic variability associated with specific pathologies such as ADPKD. Consequently, disease-tailored models trained specifically on pathologic kidneys appear necessary to achieve reliable segmentation performance and accurate morphologic assessment in this patient population.

Similar to the present work, a number of CT-based deep learning approaches have been developed for automated kidney segmentation and TKV quantification in ADPKD. Sharma et al [45] used a fully convolutional network and reported a DSC of 0.86, while Shin et al [46] employed a V-Net architecture for kidney and liver segmentation, achieving a DSC of 0.96. More recently, Sheng et al [47] combined SSD Inception V2 and DeepLabV3+ for localization and segmentation on contrast-enhanced and noncontrast CT, reporting DSC values of 0.95–0.96, while Keshwani et al [48] used a multi-task 3D FCN, achieving DSC of 0.95. While these studies demonstrated excellent segmentation accuracy and TKV estimation, they were limited to cross-sectional analyses. In contrast, our study additionally evaluates longitudinal CT examinations to assess disease progression and extends the automated workflow to MIC assignment, providing direct translation of segmentation results into a clinically established risk stratification tool.

Although the proposed study has several limitations. Our training dataset originated from a single institution, covering a relatively narrow population. This could drive the DL model to learn biases related to the studied cohort, which may limit its generalizability. Although external validation suggested good generalizability, a larger multicenter study could help in assessing the performance more accurately. Additionally, both test cohorts in our study were not particularly large, which may affect both the segmentation results and the MIC assignment. The lack of significant changes in MIC between scans should therefore be interpreted with caution, as the observed stability may reflect limited statistical power rather than a true absence of change, particularly given that one patient moved up and one moved down in MIC class. Furthermore, follow-up intervals were not standardized, which may affect the direct clinical applicability of our findings. Another important limitation relates to the reference standard used in this study. The ground truth segmentations were generated by a junior radiologist under the supervision of a senior abdominal radiologist, but no independent multi-reader annotations or inter-rater variability analysis were performed. As kidney segmentation in ADPKD can be particularly challenging due to distorted anatomy, large cyst burden, and poorly defined organ boundaries, the absence of an inter-observer agreement assessment may limit the robustness of the reference standard. Additionally, the absence of Mayo class 1A patients in the test cohort implies that the proposed framework was not evaluated in very early or mild disease stages. However, this likely reflects clinical practice, where patients with near-normal kidney size are typically assessed using ultrasound rather than CT-based volumetry. Separate segmentation and volume quantification of cystic *versus* renal parenchyma is of high clinical importance [28, 29], something that our study did not address. Such a limitation relates to the fact that this task requires a tedious manual annotation process, which is not possible in CT scans without contrast administration, but rather feasible only in MRI or contrast-enhanced CT.

In conclusion, the proposed approach can calculate TKV and renal axes in patients with ADPKD accurately, reliably, and promptly. Based on these, automated MIC assignment is straightforward, making our method a promising tool to help and guide radiologists in their clinical decision-making. Given the significant speed-up in the segmentation and MIC assignment process (around 16–18 s), our approach holds potential for direct integration into the clinical workflow. Such a DL-based method could be seamlessly integrated within the hospital’s PACS system, facilitating the automated prediction of CT images acquired with a standard ADPKD protocol. Nevertheless, a radiologist-in-the-loop remains essential to verify the automated segmentation results, particularly in cases involving very large kidneys where segmentation accuracy may decrease and manual correction may be required.

## Supplementary information

**Additional file 1: Table 1.** Training set patient characteristics. **Figure 1.** Distribution of DSC for the in-house (left) and TotalSegmentator (right) models on the test set of 60 cases (2 consecutive scans of 30 patients).

## Data Availability

The datasets generated and/or analyzed during the current study are not publicly available due to patient privacy and institutional restrictions. However, the trained model weights are available from the corresponding author upon reasonable request.

## Abbreviations

3D: Three-dimensional
ADPKD: Autosomal dominant polycystic kidney disease
CI: Confidence interval
CT: Computed tomography
DSC: Dice similarity coefficient
eGFR: Estimated GFR
GFR: Glomerular filtration rate
HtTKV: Height-adjusted total kidney volume
ICC: Intraclass correlation coefficient
KD: Kidney depth
KL: Kidney length
KW: Kidney width
MIC: Mayo imaging classification
MRI: Magnetic resonance imaging
TKV: Total kidney volume
