# Supplementary material for: Assessment of autosomal dominant polycystic kidney disease severity using deep learning–based kidney volumetry in CT
Source: Eur Radiol Exp. 2026 Nov 12;10:176. doi: 10.1186/s41747-026-00840-w (PMC13759991; doi:10.1186/s41747-026-00840-w)
Supplement: Supplementary file 1 [file 41747_2026_840_MOESM1_ESM.docx]

**Training set characteristics**

Table 1 demonstrates patients characteristics for the entire training cohort. Patients were scanned on various Siemens CT scanners using a reference kV of 120 and a quality reference mAs of 171. The collimation was set at 128x0.6 mm, with a pitch of 0.6 and a rotation time of 0.5 seconds. Images reconstructed using a soft tissue reconstruction kernel were utilized.

| **Table 1. Training set patient characteristics** | | | |
| --- | --- | --- | --- |
|  | **Training cohort, n=60** | **Scan** | |
|  | Age, years | 49.50 (31.75 – 52.00) | |
|  | Sex, men:women | 30:30 | |
|  | CTDIvol, mGy | 10.00 (4.65) | |
|  | Scanner | SIEMENS | SOMATOM Force, Definition Edge, Flash |
|  |  | TOSHIBA | Aquillion ONE |
|  |  | Philips | Ingenuity Core |
|  | Pixel spacing, mm | 0.71 (0.12) | |
|  | Slice thickness, mm | 3 | |
|  | TKV, mL | 882.28 (793.57) | |
|  | KL, mm | 179.00 (50.83) | |
|  | KW, mm | 111.69 (30.84) | |
|  | KD, mm | 92.62 (32.21) | |

**Correlation of HtTKV, HtKL, HtKW and HtKD with eGFR**

Both manual and automated measurements of HtTKV, HtKL, HtKW, and HtKD correlated significantly and negatively with eGFR. For scan 1, Pearson’s correlation values between manual measurements and eGFR were: HtTKV (-0.38), HtKL (-0.40), HtKW (-0.44), and HtKD (-0.62) with *p* < 0.001. For scan 2, similar negative correlations were observed: HtTKV (-0.42), HtKL (-0.49), HtKW (-0.52), and HtKD (-0.62) with *p* < 0.001. For the automated measurements, the Pearson’s correlation values with eGFR were HtTKV (-0.44), HtKL (-0.42), HtKW (-0.48), and HtKD (-0.59) for scan 1 and HtTKV (-0.48), HtKL (-0.47), HtKW (-0.52), and HtKD (-0.60) for scan 2, with *p* < 0.001.

**TotalSegmentator validation**

Comparison of the DSC distribution between the in-house tool and TotalSegmentator was statistically significant (*p* < 0.001). Figure 1 demonstrates the DSC distributions of both methods.

Figure 1. Distribution of DSC for the in-house (left) and TotalSegmentator (right) models on the test set of 60 cases (2 consecutive scans of 30 patients).
